# Primary cellulitis and cutaneous abscess caused by *Yersinia enterocolitica* in an immunocompetent host: A case report and literature review

**DOI:** 10.1097/MD.0000000000003988

**Published:** 2016-07-01

**Authors:** Hirofumi Kato, Shugo Sasaki, Noritaka Sekiya

**Affiliations:** Department of Clinical Laboratory, Tokyo Metropolitan Cancer and Infectious Diseases Center Komagome Hospital, Tokyo, Japan.

**Keywords:** antibiotics, drainage, primary, skin and soft-tissue infection, yersiniosis

## Abstract

Primary extraintestinal complications caused by *Yersinia enterocolitica* are extremely rare, especially in the form of skin and soft-tissue manifestations, and little is known about their clinical characteristics and treatments. We presented our case and reviewed past cases of primary skin and soft-tissue infections caused by *Y enterocolitica*. We report a case of primary cellulitis and cutaneous abscess caused by *Y enterocolitica* in an immunocompetent 70-year-old woman with keratodermia tylodes palmaris progressiva. She presented to an outpatient clinic with redness, swelling, and pain of the left ring finger and left upper arm without fever or gastrointestinal symptoms 3 days before admission. One day later, ulceration of the skin with exposed bone of the proximal interphalangeal joint of the left ring finger developed, and cefditoren pivoxil was described. However, she was admitted to our hospital due to deterioration of symptoms involving the left finger and upper arm. Cefazolin was initiated on admission, then changed to sulbactam/ampicillin and vancomycin with debridement of the left ring finger and drainage of the left upper arm abscess. Wound culture grew *Y enterocolitica* serotype O:8 and methicillin-sensitive *Staphylococcus aureus*. Blood cultures were negative and osteomyelitis was ruled out. Vancomycin was switched to ciprofloxacin, then skin and soft-tissue manifestations showed clear improvement within a few days. The patient received 14 days of ciprofloxacin and oral amoxicillin/clavulanate and has since shown no recurrence. We reviewed 12 cases of primary skin and soft-tissue infections caused by *Y enterocolitica* from the literature. In several past cases, portal entry involved failure of the skin barrier on distal body parts. Thereafter, infection might have spread to the regional lymph nodes from the ruptured skin. *Y enterocolitica* is typically resistant to aminopenicillins and narrow-spectrum cephalosporins. In most cases, these inefficient antibiotic agents were initially prescribed, but patient conditions rapidly improved after implementing appropriate therapy and drainage. In addition, primary skin and soft-tissue infections occurred even in patients lacking risk factors. Physicians should consider the rare differential diagnosis of *Y enterocolitica* infection when seeing patients with deteriorating skin lesions under standard treatment, even if the patient is immunocompetent.

## 1 Introduction

Members of the genus *Yersinia* are Gram-negative coccobacilli, representing facultative anaerobes from the family Enterobacteriaceae.^[1]^ The genus *Yersinia* includes 11 species, one of which is *Y enterocolitica*, which is known to cause gastroenteritis through contaminated food or water, or rarely via transfusion. On the other hand, septicemia from acute yersiniosis can occur in immunocompromised hosts and develop various secondary complications, such as abscesses.^[1,2]^ However, primary extraintestinal complications are extremely rare, especially in the form of skin and soft-tissue manifestations, and little is known about their clinical characteristics and treatments. We report herein a case of primary cellulitis and cutaneous abscess caused by *Y enterocolitica* in an immunocompetent host, which was successfully treated with the combination of appropriate antibiotics and surgical drainage. We discuss this case with reference to the literature.

## 2 Case presentation

A healthy, 70-year-old woman presented to an outpatient clinic with redness, swelling and pain of the left ring finger and left upper arm 3 days before her admission. She did not report experiencing any fever, chills, or gastrointestinal symptoms. Her past medical history included keratodermia tylodes palmaris progressiva and bronchial asthma without medications. The patient had a dog and denied any recent travel, blood transfusion, or consumption of raw pork. She also had a history of current smoking and social drinking. Her occupation was being a housewife. One day later, ulceration of skin with exposure of the bone at the proximal interphalangeal joint developed on the left ring finger, and cefditoren pivoxil was described. However, her condition deteriorated, and she was referred and admitted to our hospital. On admission, she had no fever, and her vital signs were stable. Physical examination revealed discharge of pus and fistulization of the left finger, and subcutaneous redness, swelling and pain of the left upper arm. The peripheral white cell blood count was 12,000 cells/mm^3^ and C-reactive protein level was 4.51 mg/dL on admission. Other blood counts, electrolytes, liver enzymes, and renal functions showed normal range. X-ray and magnetic resonance imaging (MRI) of the left hand showed no osteomyelitis.

On the day of admission (hospital day 1), cefazolin was initiated after taking blood and wound cultures. On hospital day 2, incision and drainage of the left ring finger were performed. Empiric therapy (sulbactam/ampicillin and vancomycin) were initiated, targeting skin flora including anaerobes. Wound culture grew *Y enterocolitica* and methicillin-sensitive *Staphylococcus aureus* (MSSA), and cultures of the operative specimen and abscess from the left upper arm grew only *Y enterocolitica*. Blood cultures yielded negative results. The O:8 serotype of *Y enterocolitica* was confirmed using a slide agglutination test. The isolate showed sensitivity to trimethoprim-sulfamethoxazole, aminoglycosides, tetracycline, third-generation cephalosporins, and quinolones (Table 1). Vancomycin was therefore discontinued and ciprofloxacin was started. Sulbactam/ampicillin was continued for MSSA and anaerobe coverage, although it was indistinguishable whether MSSA, which was grown in the wound culture, was the causative pathogen or the normal bacteriae on the skin. Skin and soft-tissue manifestations clearly improved after initiating ciprofloxacin, and she was discharged on hospital day 13 after changing pharmacotherapy to oral amoxicillin/clavulanate and ciprofloxacin. The patient received a total of 14 days of these antibiotic treatments without side effect and has shown no recurrence as of the time of writing.

**Table 1:**
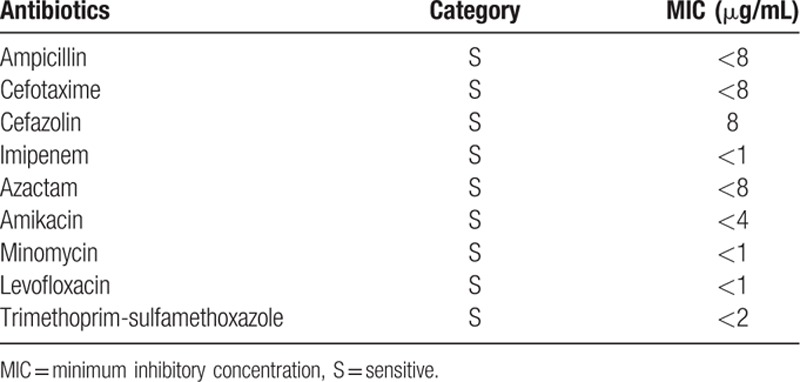
Antibiotic susceptibility profile of *Yersinia enterocolitica* isolated from our patient.

## 3 Methods

We reviewed past cases of primary skin and soft-tissue infections, including cellulitis or cutaneous abscess, caused by *Y enterocolitica* by conducting a PubMed (http://www.ncbi.nlm.nih.gov/pubmed; accessed December 3, 2015) search using the terms “*Yersinia enterocolitica*” combined with “cellulitis” or “skin infection,” “soft-tissue infection,” “cutaneous infection” from 1969 to 2014. We excluded secondary skin and soft-tissue infections by *Y enterocolitica*, which were defined by accompanying septicemia or gastrointestinal symptoms. We identified 6 articles (8 cases) that satisfied these criteria, and included an additional 4 cases cited in these articles (Table 2).

**Table 2:**
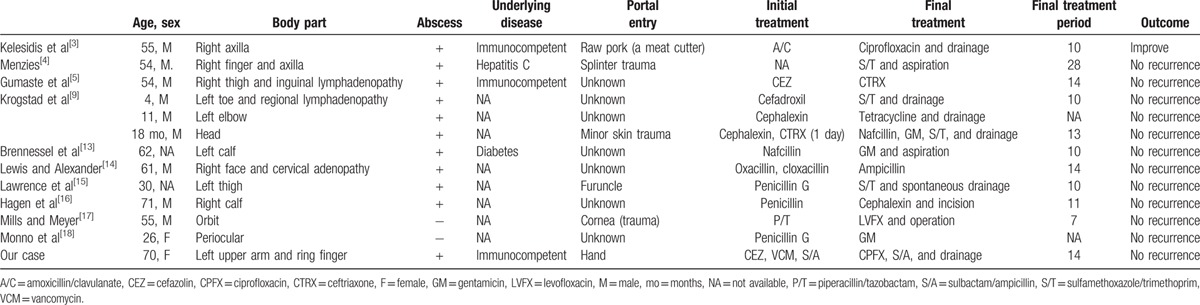
Clinical characteristics of cases of primary skin and soft-tissue infections caused by *Yersinia enterocolitica* (n = 12).

### 3.1 Ethics

We obtained informed consent from the patient. In addition, this manuscript was a case report. Thus, this did not require approval from an ethics committee.

## 4 Discussion

We encountered a case of primary cellulitis and cutaneous abscess caused by *Y enterocolitica* in an immunocompetent host, which appear to represent rare manifestations. Furthermore, a first-generation cephalosporin and aminopenicillin were not clinically effective despite the susceptibility profiles of the isolated strain, and surgical drainage proved necessary.

Yersiniosis usually presents with gastrointestinal symptoms. On the other hand, most extraintestinal manifestations of *Y enterocolitica* have been described as a consequence of septicemia among individuals with impaired immunity.^[1,2]^ Abscess formation is also usually caused by septicemia in immunocompromised hosts.^[3–5]^ Our case showed no evidence of septicemia or gastrointestinal symptoms, so the pathogen was inferred to have directly entered into the tissue from some environmental source through a weakened skin barrier. In several past cases, portal entry also involved a failure of the skin barrier. In particular, most lesions were observed in distal body parts, including the extremities and face where injuries readily occur. Further, infection might spread to the regional lymph nodes after entering from areas of ruptured skin.^[4]^ Physicians should therefore pay attention to the distal parts and associated regional lymph nodes, including abscess formation. In addition, risk factors for *Yersinia* bacteremia are considered to include liver disease, hemochromatosis, iron overload, malignancy, and diabetes mellitus.^[6]^ However, our review of the literature did not find these risk factors in most cases, nor were they present in our own case. This means that primary skin and soft-tissue infections can occur even in patients not showing these risk factors.

This case was successfully treated with the combination of appropriate antibiotics and surgical drainage. Antibiotics are not always needed for gastrointestinal infections, which are self-limiting diseases. However, they are warranted for extraintestinal infections including skin and soft-tissue infections, septicemia, and immunocompromised status.^[1]^ It is important for physicians that *Y enterocolitica* isolates are typically resistant to the antibiotics often in general use for skin and soft-tissue infections, such as first-generation cephalosporins and most penicillins, even if the organisms show antibiotic susceptibility.^[7]^ Isolates are usually susceptible to aminoglycosides, tetracycline, trimethoprim-sulfamethoxazole, third-generation cephalosporins, and quinolones.^[8]^ In addition, most cases deteriorated on first-generation cephalosporins and most penicillins. In this case, although cefditoren pivoxil, cefazolin, and sulbactam/ampicillin were described before and after admission with surgical drainage, insufficient clinical improvement of skin lesions was achieved despite the susceptibility profile of the isolate. In cases with abscess formation, surgical drainage should be considered in addition to antibiotics.^[9]^ As soon as ciprofloxacin was added after identifying *Y enterocolitica* from drainage, clear improvements were achieved within a few days. Most past cases have also improved after implementing effective antibiotics and surgical therapy, and no recurrences were identified.

Organisms of the O:3, O:8, and O:9 serotypes are the most frequent causes of sporadic human disease worldwide.^[10]^ In Japan, the most common serotypes are O:3 and O:8, which cause sporadic illness and occasional food-borne outbreaks.^[11]^ Serogroup O:8 may cause enteritis or mesenteric lymphadenitis. This strain has mainly been associated with zoonotic reservoirs.^[1]^ We initially suspected transmission from raw meat, but the patient had no history of contacts or eating raw meat. The household dog in this case was suspected as another potential route of transmission, because *Y enterocolitica* is reportedly related to exposure to household dogs.^[12]^ We examined a stool culture from the patient's dog, but results were negative. The route of pathogen transmission thus remained unclear.

This case involved primary cellulitis and cutaneous abscess caused by *Y enterocolitica* in an immunocompetent host, and successfully treated with the combination of antibiotics and surgical drainage. Physicians should consider the rare differential diagnosis of *Y enterocolitica* infection when seeing patients with deteriorating skin lesions under standard treatment, even if the patient is immunocompetent.
